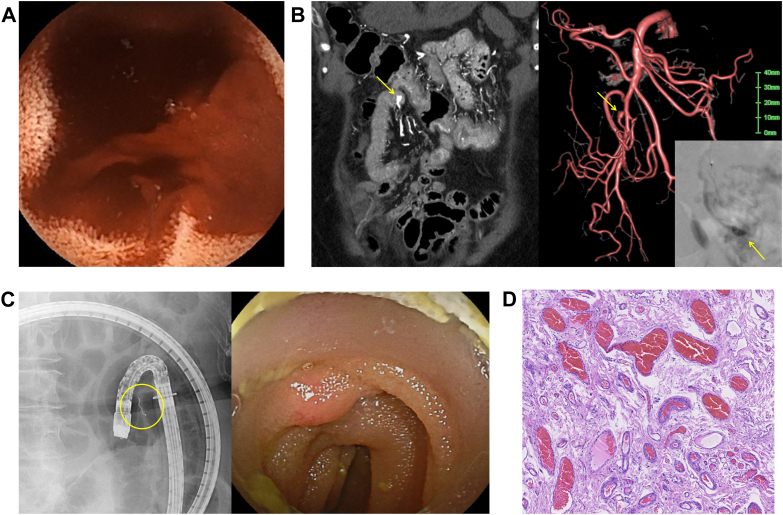# Obscure Recurrent Upper Gastrointestinal Bleeding Caused by a Jejunal Arteriovenous Malformation

**DOI:** 10.1016/j.gastha.2025.100877

**Published:** 2025-12-30

**Authors:** Masahiro Yanagi, Hajime Takatori, Taro Yamashita

**Affiliations:** Department of Gastroenterology, Kanazawa University Hospital, Kanazawa, Ishikawa, Japan

A 75-year-old woman with liver cirrhosis was referred for recurrent melena and dyspnea. Despite repeated upper and lower gastrointestinal endoscopy, contrast-enhanced computed tomography, and bleeding scintigraphy, no bleeding source was identified. One month later, capsule endoscopy revealed active bleeding from the upper jejunum (Figure A). Emergency balloon-assisted enteroscopy failed to localize the lesion, and the bleeding resolved temporarily. Six months later, melena recurred. Selective angiography demonstrated a mass-like structure protruding into the jejunal lumen from a branch of the superior mesenteric artery (Figure B). Coil embolization was performed; however, bleeding recurred again 1 month later. Double-balloon endoscopy using the embolized coil as a landmark revealed a 5-mm submucosal tumor-like prominence with overlying erythema in the upper jejunum (Figure C). Surgical resection was subsequently performed. Histopathological examination demonstrated dilated and irregular arteriovenous channels within the submucosal layer, consistent with jejunal arteriovenous malformation (AVM; Figure D). The postoperative course was uneventful. Endoscopically, gastrointestinal AVMs typically appear as low-profile submucosal tumor-like elevations, sometimes accompanied by erythema, erosion, or pulsation. However, these findings are often subtle or transient, making diagnosis challenging. This case highlights the importance of multimodal evaluation for identifying small bowel AVMs in patients with obscure gastrointestinal bleeding.

**Figure:**